# Comparative evaluation of the therapeutic efficacy between human amniotic epithelial cells and human umbilical cord mesenchymal stem cells in premature ovarian insufficiency

**DOI:** 10.1186/s13287-025-04881-7

**Published:** 2025-12-31

**Authors:** Qinyu Zhang, Jie Wang, Zixin Cheng, Wenjiao Cao, Qiuwan Zhang, Dongmei Lai

**Affiliations:** 1 The International Peace Maternity and Child Health Hospital, School of Medicine, Shanghai Jiao Tong University, Hengshan Road 910, Shanghai, 200030 China; 2 Shanghai Key Laboratory of Embryo Original Diseases, Guangyuan Road 145, Shanghai, 200030 China

**Keywords:** Premature ovarian insufficiency, Human amniotic epithelial cells, Human umbilical cord mesenchymal stem cells, Metabolic kinetics, Paracrine factors

## Abstract

**Background:**

Premature ovarian insufficiency (POI) is a clinically challenging condition characterized by amenorrhea and infertility in women less than 40 years of age. Although both human amniotic epithelial cells (hAECs) and human umbilical cord mesenchymal stem cells (hUC-MSCs) have shown promise in treating POI, their comparative therapeutic efficacy and mechanisms remain poorly understood.

**Methods:**

hAECs and hUC-MSCs were isolated from human amniotic membrane and umbilical cords, respectively, and characterized using standard protocols. A chemotherapy-induced POI mouse model was established to evaluate follicular development, ovarian fibrosis, and fertility recovery after hAEC and hUC-MSC transplantation. Longitudinal in vivo bioluminescence imaging was used to track and compare the biodistribution and retention rates of the transplanted cells. RNA sequencing and in vitro functional assays under oxidative stress and apoptosis-induced conditions were employed to analyze the differential stress responses of hAECs and hUC-MSCs. Furthermore, cytokine arrays were utilized to profile their secretomes.

**Results:**

In the chemotherapy-induced POI mouse model, both hAECs and hUC-MSCs transplantation improved ovarian function, as evidenced by increased ovarian weight, restored estrous cycle, elevated follicle counts, reduced fibrosis, and enhanced fertility. In vivo imaging revealed that both cell types primarily homed to the lungs, liver, and spleen post-transplantation, with signal intensity declining over time. Quantitative analysis revealed significantly longer in vivo retention of hAECs compare to hUC-MSCs. RNA sequencing and in vitro assays confirmed the superior antioxidant capacity of hAECs under stress conditions. Cytokine profiling showed that hAEC-CM was enriched in pro-angiogenic factors, while hUC-MSC-CM contained higher levels of immunoregulatory cytokines, a functional difference further validated by in vitro experiments.

**Conclusion:**

Our findings demonstrate that both hAECs and hUC-MSCs are effective in restoring ovarian function and fertility in a chemotherapy-induced POI mouse model. However, these two cell types exhibit distinct therapeutic advantages attributable to their differential metabolic kinetics and paracrine profiles. Specifically, hAECs displayed prolonged in vivo retention rates compared to hUC-MSCs, consistent with their enhanced antioxidant capabilities. In terms of secretory function, hAECs demonstrated superior pro-angiogenic activity, while hUC-MSCs exhibited stronger immunomodulatory effects. These distinct properties provide critical insights for cell-type-specific selection in developing targeted therapies for ovarian dysfunction.

**Supplementary Information:**

The online version contains supplementary material available at 10.1186/s13287-025-04881-7.

## Background

POI is a devastating endocrine disorder affecting 1–3% of women under 40 years of age worldwide [1]. Characterized by the depletion of ovarian follicle before the age of natural menopause, POI presents with key biochemical features including hypergonadotropism (FSH > 25 IU/L), hypoestrogenemia and amenorrhea. Beyond its reproductive implications, POI is also associated with multisystem metabolic disorders, an increased risk of age-related comorbidities, and neurocognitive deficits, collectively significantly endangering women’s quality of life, and physical and mental health [2].

Chemotherapy-induced POI is a serious iatrogenic complication that affects 40–80% of reproductive-age cancer survivors, with alkylating agents showing pronounced gonatotoxicity [3]. The mechanisms of chemotherapy-induced POI mainly include DNA cross-link formation in oocytes or granulosa cells, leading to apoptosis, and the excessive activation and subsequent exhaustion of dormant primordial follicles [4]. In addition, the ovarian microenvironment undergoes severe remodeling, marked by stromal fibrosis, collapse of cortical vasculature, and mitochondrial dysfunction in oocytes [5].

Hormone replacement therapy (HRT), the standard treatment involving exogenous estrogen and progesterone supplementation, has a fundamentally limited therapeutic scope as it neither reverses follicular atresia nor restores ovarian endocrine cycles. Although embryo, oocyte, and ovarian tissue cryopreservation have been established as effective and feasible methods for fertility protection, they also face numerous limitations [6]. This urgent unmet clinical need highlights the necessity of developing novel interventions capable of repairing ovarian function.

Recent encouraging findings have facilitated the initiation of numerous clinical trials utilizing stem cells for treating various diseases [7]. Stem cell therapy has emerged as a transformative strategy for ovarian regeneration, capitalizing on its distinctive capacity to orchestrate tissue regeneration repair through multi-target and multi-level mechanisms [8]. Moreover, substantial evidence indicates that stem cell transplantation can restore follicular development, activate dormant primordial follicles, and reestablish endocrine balance, thereby offering promising therapeutic potential for POI [9].

hAECs and hUC-MSCs are two distinct types of perinatal stem cell with unique biological properties and therapeutic potential [10]. Originating from the umbilical cord, hUC-MSCs are mesenchymal lineage cells known for their robust proliferative capacity and tri-lineage differentiation potential [11]. hAECs, derived from the epithelial layer of amniotic membrane, exhibit embryonic-like pluripotent markers and have demonstrated promising therapeutic effects in numerous studies across various disease [12, 13]. These intrinsic multilineage differentiation capabilities, along with their accessibility, establish them as preeminent cell sources for regenerative therapy.

An increasing number of studies indicates that hUC-MSCs restore ovarian function by activating multiple pathways, including promoting angiogenesis, immune modulation and inhibiting apoptosis [14, 15]. The application of hUC-MSCs for POI treatment has advanced to clinical trials, indicating that hUC-MSCs transplantation can enhance follicular development; improve ovarian function and fertility outcomes in patients [16]. In animal models of POI, hAECs have exhibited significant therapeutic benefits, with studies elucidating potential mechanisms, such as cell trans-differentiation, stimulation of follicular development, and restoration of the damaged ovarian microenvironment [17]. Recently, a phase I clinical trial involving hAECs delivered by ovarian arterial injection was completed in POI patients, showing favorable safety and efficacy [18]. However, the comparative therapeutic potential between hAECs and hUC-MSCs in ovarian function repair remains inadequately explored.

To compare the therapeutic potential differences between hAECs and hUC-MSCs in restoring ovarian function, we evaluated ovarian function and fertility outcomes in a mouse model of POI. In vivo imaging was used to track the biodistribution and metabolic activity of engrafted hAECs and hUC-MSCs. Subsequently, in vitro assays were conducted to assess their proliferative, antioxidant, and anti-apoptotic capacities. Furthermore, cytokine array profiling was performed to systematically compare their paracrine secretion patterns.

## Methods

### Isolation and culture of hAECs and hUC-MSCs

hAECs were isolated from placentas obtained from HIV/hepatitis B/C-negative donor following our previously established protocol [19]. Briefly, the amniotic membrane was mechanically separated from the chorion, thoroughly washed with phosphate-buffered saline (PBS, Gibco), and digested with 0.25% trypsin-EDTA (Gibco) at 37 °C for 25 min. The cell suspension was sequentially filtered through a 40-µm cell strainer (Corning) and centrifuged at 300×*g* for 5 min. The cell pellet was resuspended in DMEM/F12 medium (Gibco) supplemented with 10% fetal bovine serum (FBS, Gibco), 2mM glutamine (Gibco), and 100 U/mL penicillin, and 100 µg/mL streptomycin (HyClone). Cells were maintained at 37 °C in a humidified atmosphere containing 5% CO₂, with medium replenished every 72 h until they reached 80% confluence. hAECs at passages 2–4 were used for all subsequent experiments.

hUC-MSCs were isolated following previously described protocol [20]. Briefly, umbilical cord tissue collected from donors was dissected after removal of the arteries and veins, and divided into 2–3 mm³ explants. These explants were cultured in a basal medium (Stemcell Technologies) under standard conditions (37 °C, 5%CO_2_, humidified atmosphere). A primary cell outgrowth with a characteristic fibroblast-like morphology was observed, and the original explants were removed. The cells were then enzymatically dissociated using 0.25% Trypsin-EDTA and subsequently resuspended in complete medium and seeded. hUC-MSCs at passages 2–4 were used for further experiments.

### Identification of hAECs and hUC-MSCs

hAECs and hUC-MSCs were characterized using flow cytometry and immunofluorescence. For flow cytometric analysis, cells were harvested, washed, and stained with the following fluorescently conjugated primary antibodies: CD324, CD34, CD73, CD90 and HLA-DR (all from BioLegend, San Diego, CA, USA) for 30 min at 4 °C in the dark. After staining, cells were washed with cold PBS and analyzed immediately using a FC500 flow cytometer (Beckman Coulter).

For immunofluorescence characterization, cells were fixed with 4% paraformaldehyde (PFA, Boster Biological Technology) for 15 min at room temperature. After permeabilization with 0.1% Triton X-100 (Sigma-Aldrich, St. Louis, MO, USA), cell were incubated with the following primary antibodies at 4 °C for overnight: OCT4 (1:200, Boster), CK18 (1:200, Boster) and N-cadherin (1:200, Boster). Cells were then incubated with Alexa Fluor 488-conjugated secondary antibody (1:1000, Thermo Fisher Scientific) for 1 h at room temperature. Nuclei were counterstained with DAPI (1:1000, Sigma-Aldrich), and fluorescence signals were visualized using a confocal microscope (Leica, Wetzlar, Germany).

### Adipogenic, osteogenic, and chondrogenic differentiation of hUC-MSCs

To evaluate the multipotent differentiation potential of hUC-MSCs, a commercial multiplex induction kit (Absin, Shanghai, China) was used for adipogenic, chondrogenic, and osteogenic differentiation. For adipogenic differentiation, cells were subjected to cyclic culture using induction and maintenance media according to the manufacturer’s protocol for 10 days, followed by Oil Red O staining. Osteogenic differentiation was induced for 2 weeks, and mineralization was evaluated by Alizarin Red S staining. For chondrogenic differentiation, cells were cultured as aggregates for 2 weeks. The resulting aggregates were embedded in Tissue-Tek O.C.T. compound (Sakura, Japan), sectioned, and stained with Alcian blue following the kit instructions.

### Establishment of animal models

Female C57BL/6J mice aged 6–8 weeks were purchased from Shanghai Lingchang Bio and housed under a 12 h light/dark cycle at 22℃ with unrestricted access to food and water. The study was approved by the Institutional Animal Care and Use Committees of International Peace Maternal and Child Health Hospital. Every effort was made to alleviate animal suffering and reduce the number of animals used. All animal experiments adhere to the ARRIVE guidelines 2.0.

A total of 76 female C57/BL6 mice (18–20 g) were randomly divided into the chemotherapy-induced POI group (Cy; *n* = 53) and a sham control group (Sham, *n* = 23). POI were induced via a single intraperitoneal injection of busulfan (30 mg/kg, Sigma-Aldrich) and cyclophosphamide (120 mg/kg, Hengrui, Jiangsu, China), as previously described [21]. Sham group mice were injected with an equal volume of PBS.

### Ovarian function and fertility evaluation

Forty five mice in Cy group were randomly divided into three subgroups, including hAEC transplantation group, hUC-MSC transplantation group and PBS control group. Mice in hAECs-transplanted group (Cy + hAECs; *n* = 15) were injected with hAECs (2 × 10^6^ cells in a volume of 150 µL PBS) by tail vein at Day 7 after chemotherapy. Mice in hUC-MSCs-transplanted group (Cy + hUC-MSCs; *n* = 15) were injected with hUC-MSCs (2 × 10^6^ cells in a volume of 150 µL PBS) by tail vein at Day 7 after chemotherapy. Mice in PBS control group (Cy; *n* = 15) were injection with PBS (150 µL) by tail vein at Day 7 after chemotherapy.

In the fertility assessment experiment, the mating experiment was conducted 1 week after stem cell transplantation. Female mice from different treatment groups (*n* = 5 per group) were paired with fertile males. Fertility was assessed by examining embryonic development at 16.5–17.5 days after a vaginal plug was detected. Briefly, pregnant mice were anesthetized via an intraperitoneal injection of tribromoethanol (20 µL/g, Beran, China), and the number and morphology of embryos in their uteri were examined.

For ovarian morphological evaluation and hormone measurement, blood and ovarian tissue samples were collected on days 14 and 28 post-transplantation (*n* = 5 mice per group per time point). Briefly, blood samples were collected from the orbital sinus of mice anesthetized with tribromoethanol. Following blood collection, euthanasia was confirmed by cervical dislocation, and ovarian tissues were harvested for subsequent analysis.

### Vaginal smear procedure and estrous cycle phase analysis

Vaginal smears were performed for 8 consecutive days. At 9:00 a.m., sterile saline solution was heated to 40 °C and carefully inserted into the vagina using a pipette. Drops containing vaginal smears were mounted on glass slides, stained with Giemsa Solution and observed under a light microscope. The estrous cycle stage of each mouse was determined independently by two researchers in accordance with established morphological criteria [22].

### In vivo tracking of hAECs and hUC-MSCs

hAECs and hUC-MSCs were transduced with a lentiviral vector carrying luciferase and GFP reporter genes (GL161 pASLenti-pA-Luc2-CMV-EF1-EGFP-P2A-Puro- WPRE, Obio Technology, Shanghai, China). Transduction efficiency exceeded 90% as confirmed by fluorescence microscopy. Subsequently, puromycin (5 µg/mL, Yeasen, Shanghai, China) was applied for 72 h to select for GFP-positive cells.

Lentivirus-transduced hAECs or hUC-MSCs (2 × 10^6^ in 150 µL PBS per mouse) were administrated via tail vein injection to both sham mice (Sham + hAECs group and Sham + hUC-MSCs group, *n* = 4 per group) and chemotherapy-treated POI mice (Cy + hAECs group and Cy + hUC-MSCs group, *n* = 4 per group) on day 7 post-chemotherapy. Mice anesthetized with tribromoethanol were intraperitoneally injected with the luciferase substrate at 1, 2, 4 h and 1, 2, 3, 7 days after cell administration. Bioluminescence signals were subsequently detected using an in vivo imaging system at the Shanghai Model Organisms Center.

### RNA sequencing and bioinformatics analysis

RNA sequencing (RNA-seq) was performed by Oe-Biotech. Differentially expressed genes (DEGs) were calculated by log2 fold change > 1 and adjusted *p* < 0.05 by comparing the FPKM values. The biological functions of the DEGs between the two groups were analyzed by Gene Ontology (GO) classification and Kyoto Encyclopedia of Genes and Genomes (KEGG) analysis.

### RNA extraction and real-time polymerase chain reaction (PCR)

Total RNA was extracted from cells with RNAiso Plus, and then 1 µg RNA was reverse-transcribed to cDNA using RT reagent Kit. Genes of interest were amplified in QuantStudio7 Flex System with SYBR Green Real-time PCR Master Mix. PCR primers were designed according to cDNA sequences in the NCBI database. Cycling conditions for the PCR machine were as follows: 95 °C 15s and 60 °C 30s for 40 cycles. Relative mRNA expression was calculated using the 2^(−ΔΔCT)^ method normalized to GAPDH.

### Histology and immunohistochemistry analysis

Ovaries were harvested at day 28 post-transplantation of hAECs and hUC-MSCs. Tissues were fixed in 4% PFA, paraffin-embedded and serially sectioned at a thickness of 5 μm. Hematoxylin and eosin (HE) staining was used to examine ovarian morphology under a light microscopy. Follicular stages were categorized based on well-established criteria as previously described [23].

For α-SMA immunohistochemistry, ovarian sections were subjected to antigen retrieval, followed by sequential incubations with: primary antibody (rabbit anti-α-SMA, 1:2000, Servicebio, Wuhan, China), secondary antibody (biotinylated anti-rabbit IgG, 1:200; Vector Laboratories, Burlingame, CA, USA), and ABC-HRP reagent with 0.05% diaminobenzidine (DAB, Vector Laboratories) for chromogenic detection, and counterstained with hematoxylin. Negative controls omitting primary antibodies exhibited complete specificity.

### Immunofluorescence staining and analysis

Ovarian tissues collected at day 14 post-transplantation were fixed in 4% PFA, embedded in paraffin, and sectioned at 5 μm. After deparaffinization, heat-induced antigen retrieval was performed in EDTA buffer (pH 9.0) at 95 °C for 20 min. Sections were then blocked with 5% bovine serum albumin (BSA) and permeabilized with 0.3% Triton X-100. After rinsing with PBS, sections were incubated overnight at 4 °C with the following primary antibodies: anti-Ki67 (1:250, Abcam), anti-γH2AX (1:250, Abcam), anti-CD34 (1:100, Abcam), anti-Tumor necrosis factor-alpha (TNF-α, 1:250, Proteintech) and anti-Interleukin-10 (IL-10, 1:250, Proteintech). The following day, sections were incubated with appropriate fluorophore-conjugated secondary antibodies (Alexa Fluor 488 or 594, 1:1000; Abcam) for 1 h at room temperature. Nuclei were counterstained with DAPI. Fluorescence images were acquired using a confocal microscopy and signal quantification was performed with ImageJ software.

### Enzyme-linked immunosorbent assay (ELISA)

Serum levels of estradiol (E2), anti-Müllerian hormone (AMH), TNF-α and IL-10 were measured using commercial ELISA kits (CUSABIO, Wuhan, China) according to the manufacturers’ instructions.

### CCK-8 cell viability assay

hAECs and hUC-MSCs were exposed to varying concentrations of H_2_O_2_ (0, 1, 10, 100, 250, and 300 µM) and carbonyl cyanide 3-chlorophenylhydrazone (CCCP, 0, 0.1, 1, 10, and 100 µM) for 24 h. The medium was then replaced with fresh culture medium containing 10% CCK-8 reagent (Yeasen) for 2 h at 37 °C. Absorbance at 450 nm was measured using a microplate reader.

### EdU cell proliferation assay

hAECs and hUC-MSCs were treated with H_2_O_2_ (100 µM) and CCCP (10 µM) for 24 h. Cells were then fixed with 4% PFA and permeabilized with 0.5% Triton X-100. After washing with PBS, cells were incubated with the Click-iT EdU reaction mixture (Yeasen) at room temperature for 30 min. Fluorescence signals were visualized using a confocal microscope (Leica).

### TUNEL assay

Ovarian tissues collected at day 14 post-transplantation were fixed in 4% PFA, embedded in paraffin, and sectioned at 5 μm. Apoptosis was evaluated using a commercially available TUNEL assay kit (BioMed World, Shanghai, China) according to the manufacturer’s protocol, and fluorescence images were acquired with a confocal microscope.

For in vitro TUNEL assay, hAECs and hUC-MSCs treated with H₂O₂ (100 µM) and CCCP (10 µM) were fixed with 4% PFA and permeabilized with 0.2% Triton X-100. Apoptotic cells were labeled using the TUNEL kit (Yeasen), and fluorescence signals were visualized and quantified under a confocal microscope.

### SA-β-Gal staining assay

hAECs and hUC-MSCs were treated with H_2_O_2_ (100 µM) and CCCP (10 µM) for 24 h. Cells were fixed with β-galactosidase staining fixative at room temperature for 15 min, washed with PBS, and subsequently incubated with β-galactosidase staining mixture (Yeasen) at 37 °C overnight. Staining signals were imaged and quantified using a confocal microscope.

### Preparation of conditioned medium and cytokine array

Conditioned media (CM) were prepared by seeding 2 × 10⁶ hAECs or hUC-MSCs in 100 mm culture dishes. Post-adhesion, cells were serum-deprived in DMEM/F12 (Gibco) for hAECs and MSC basal medium (Stemcell Technologies) for hUC-MSCs for 24 h. The collected CM was sterile-filtered (0.22 μm, Corning), and concentrated using 3 kDa molecular weight cut-off centrifugal filters (Amicon Ultra-15, Millipore). The cytokine assay (RayBiotech AAH-CYT-G5-4) was conducted in accordance with manufacturer’s protocol.

### In vitro inflammatory response assay

RAW264.7 cells were seeded in 24-well plates and allowed to adhere overnight. Cells were then stimulated with 100 ng/mL LPS (Sigma-Aldrich) and 20 ng/mL IFN-γ (PeproTech, Wuhan, China), followed by treatment with the CM from hAECs or hUC-MSCs for 24 h. After fixation and permeabilization, cells were incubated overnight at 4 °C with anti-iNOS antibody (1:200, Proteintech), followed by incubation with Alexa Fluor 488-conjugated goat anti-rabbit IgG (1:1000, Abcam) for 1 h at room temperature. Nuclei were counterstained with DAPI, and fluorescence signals were observed using a confocal microscope.

### Western blot analysis

RAW264.7 cell lysates from hAEC-CM and hUC-MSC-CM treatment groups were separated by 12.5% SDS-PAGE and transferred onto PVDF membranes (Millipore). After blocking with 5% non-fat milk in TBST for 1 h at room temperature, membranes were incubated with primary antibodies against iNOS (1:1000, Abcam) and TNF-α (1:1000, Proteintech) at 4 °C overnight. Following TBST washes, membranes were incubated with HRP-conjugated secondary antibodies (1:3000, Proteintech). HRP-conjugated GAPDH (1:2000, Yeasen) served as the loading control. Protein bands were detected using ECL chemiluminescence substrate and imaged with an Amersham ImageQuant 800 system (Cytiva).

### In vitro tube formation assay

Human umbilical vein endothelial cells (hUVECs) were cultured in DMEM/F12 supplemented with 10% FBS. A total of 3 × 10^4^ hUVECs were seeded per well onto polymerized Matrigel (Corning) in a 24-well plate and treated with hAEC-CM or hUC-MSC-CM. After 4–6 h of incubation at 37 °C, tube-like structure was imaged using an inverted microscope. The number of branches and nodes were quantified using ImageJ software.

### Statistical analysis

All data are expressed as means ± standard error of the mean (SEM). Statistical analyses were performed using GraphPad Prism 9.0. One-way analysis of variance (ANOVA) followed by Bonferroni’s post hoc test was used for multiple group comparisons. Follicle counts and hUVECs tube formation (number of branches and nodes) were assessed independently by two blinded observers, and the average values were used for analysis. A *p*-value < 0.05 was considered statistically significant.

## Results

### Characterization of hAECs and hUC-MSCs

**Fig. 1:**
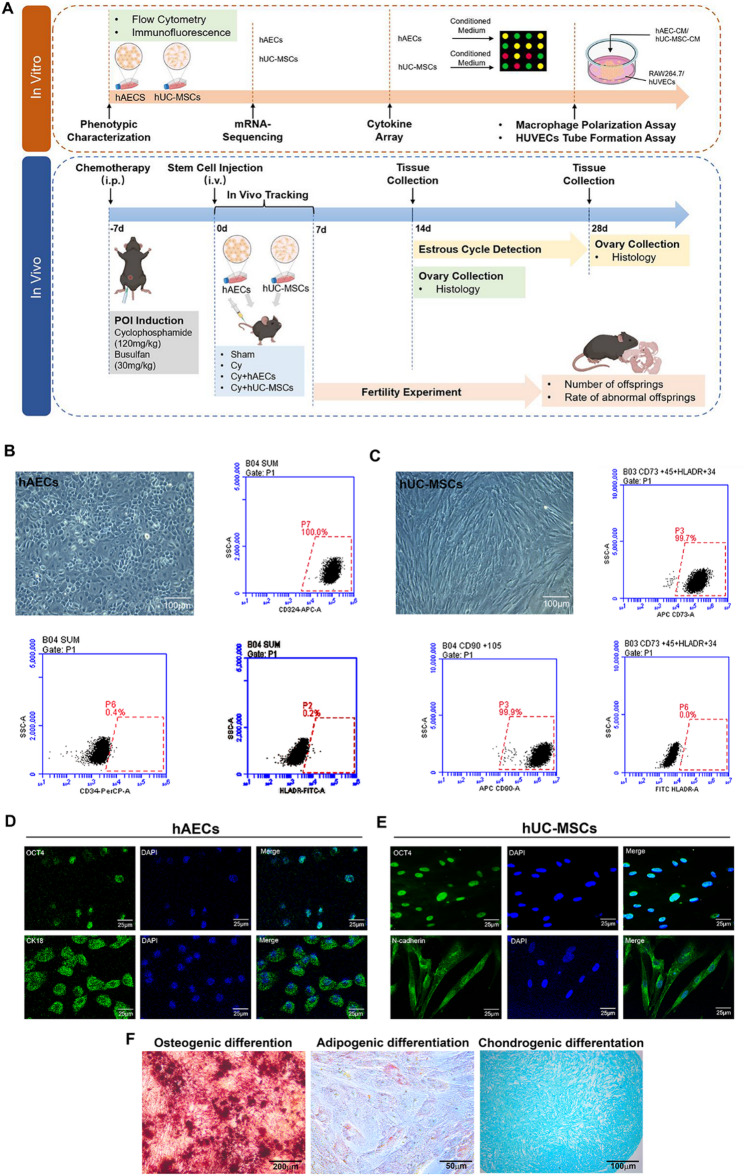
Characterization of hAECs and hUC-MSCs. **A** Schematic illustration of the experimental design for comparing the effects of hAECs and hUC-MSCs on ovarian function. **B**, **C** Morphological feature and flow cytometric analysis of hAECs and hUC-MSCs. **D**, **E** Representative images of double immunofluorescence staining for OCT4 and CK18 or N-cadherin in hAECs and hUC-MSCs. **F** Multilineage differentiation potential of hUC-MSCs into osteoblasts, adipocytes, and chondrocytes, as assessed by Alizarin Red, Oil Red O, and Alcian Blue staining, respectively. Scale bars represent 25, 50, 100 and 200 μm

hAECs and hUC-MSCs were characterized by morphological observation, flow cytometry and immunofluorescence. hAECs exhibited a typical cobblestone-like epithelial morphology, whereas hUC-MSCs showed a spindle-shaped fibroblastic appearance (Fig. 1B, C). Flow cytometric analysis confirmed the epithelial characteristics of hAECs (CD324^+^ 100%; CD34^−^; HLA-DR^−^), while hUC-MSCs displayed a standard mesenchymal immunophenotype (CD73/90^+^ >99.7%; HLA-DR^−^). Immunofluorescence staining indicated that hAECs co-expressed OCT4 and CK18, and hUC-MSCs exhibited OCT4 with N-cadherin localization (Fig. 1D, E). Multilineage differentiation assays further confirmed the adipogenic, osteogenic, and chondrogenic potential of hUC-MSCs (Fig. 1F). These results confirm the identity and purity of both hAECs and hUC-MSCs, supporting their application in subsequent experiments.

### hAEC and hUC-MSC transplantation protects ovarian function and fertility from chemotherapy-induced ovarian damage

**Fig. 2:**
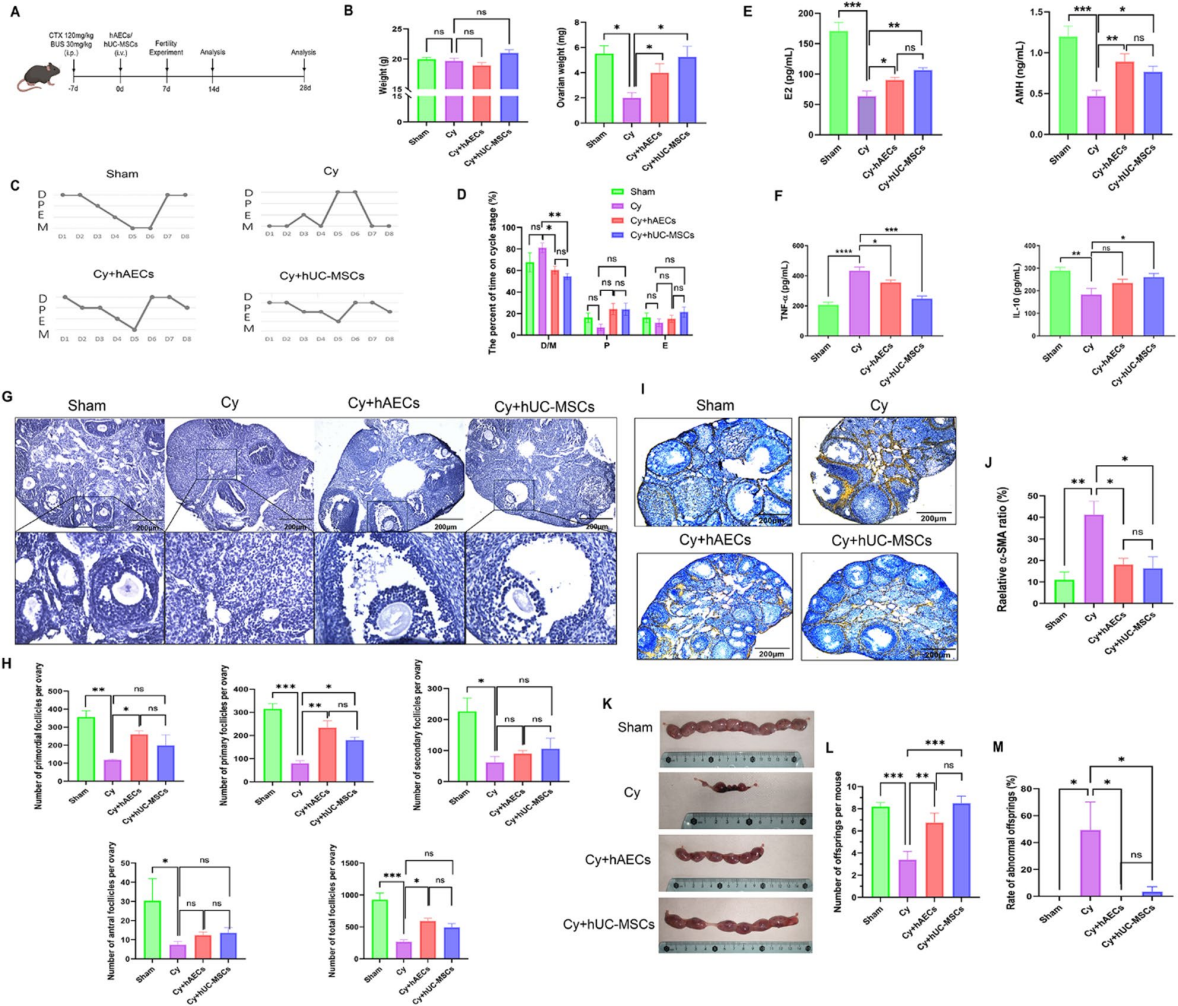
Evaluation of ovarian function and fertility in a chemotherapy-induced POI mouse model. **A** Schematic illustration of the experimental timeline and treatment groups. **B** Analysis of ovarian weight and body weight across different groups. **C** Observation of estrous cycle following hAEC and hUC-MSC transplantation. **D** Proportional distribution of each estrous cycle stage among the groups. **E** Serum levels of E2 and AMH measured by ELISA at 4 weeks post-cell transplantation. **F** Serum levels of TNF-α and IL-10 measured by ELISA at 2 weeks post-cell transplantation. **G** Histological examination of ovarian sections by HE staining. **H** Quantification of follicles at various developmental stages. (I-J) Expression levels of α-SMA in ovarian sections and expression analysis in different treatment groups. **K** Isolated uteri from pregnant mice prior to delivery. **L**, **M** Number of embryos and incidence of developmental abnormalities across treatment groups. Data are presented as means ± SEM (*n* = 5 mice per group, *P < 0.05; **P < 0.01; ***P < 0.001;****P < 0.0001). Scale bars represent 200 μm

To evaluate the therapeutic efficacy of hAECs and hUC-MSCs, we assessed their effects in a mouse model of chemotherapy-induced POI (Fig. 2A). No significant differences in body weight were observed across the groups; however, ovarian weight was markedly reduced in the Cy group relative to the Sham group. Transplantation of either hAECs or hUC-MSCs significantly increased ovarian weight compared to the Cy group (*P* < 0.05, Fig. 2B). Analysis of the estrous cycle showed an increased proportion of diestrus and metestrus phases in the Cy group, a disruption that was partly reversed by hAECs or hUC-MSCs transplantation (*P* < 0.05, Fig. 2C, D). Serum levels of E2 and AMH were significantly reduced in the Cy group, while both hAECs and hUC-MSCs transplantation increased serum E2 and AMH levels at 4 weeks post-transplantation (*P* < 0.05, Fig. 2E). In addition, serum levels of pro-inflammatory TNF-α and anti-inflammatory IL-10 at 2 weeks post-transplantation were measured. Results showed that chemotherapy caused an inflammatory response, characterized by elevated TNF-α and decreased IL-10 levels. Notably, stem cell transplantation suppressed the inflammatory response, with hUC-MSCs significantly decreasing TNF-α and elevating IL-10 levels in serum (*P* < 0.05, Fig. 2F).

Histological analysis demonstrated a significant decrease in primordial, primary, secondary, and antral follicles in the Cy group. hAEC transplantation significantly increased primordial and primary follicle numbers, while hUC-MSCs primarily restored primary follicles (*P* < 0.05, Fig. 2G, H). Additionally, ovarian fibrosis (measured by α-SMA expression) was significantly elevated in the Cy group. Both hAEC and hUC-MSC transplantation effectively reduced α-SMA expression, indicating attenuated fibrotic damage (*P* < 0.05, Fig. 2I, J). Fertility assessment showed that the Cy group produced fewer offspring and exhibited a higher incidence of developmental abnormalities compared to the Sham group. Both hAECs and hUC-MSCs significantly increased the number of embryos and reduced the rate of abnormal offspring, with no statistically significant difference observed between the two cell types (*P* < 0.05, Fig. 2K–M). These findings demonstrate that hAECs and hUC-MSCs are similarly effective in alleviating ovarian damage and restoring ovarian function in chemotherapy-induced POI.

### Effects of hAEC and hUC-MSC transplantation on chemotherapy-induced ovarian microenvironment damage

**Fig. 3:**
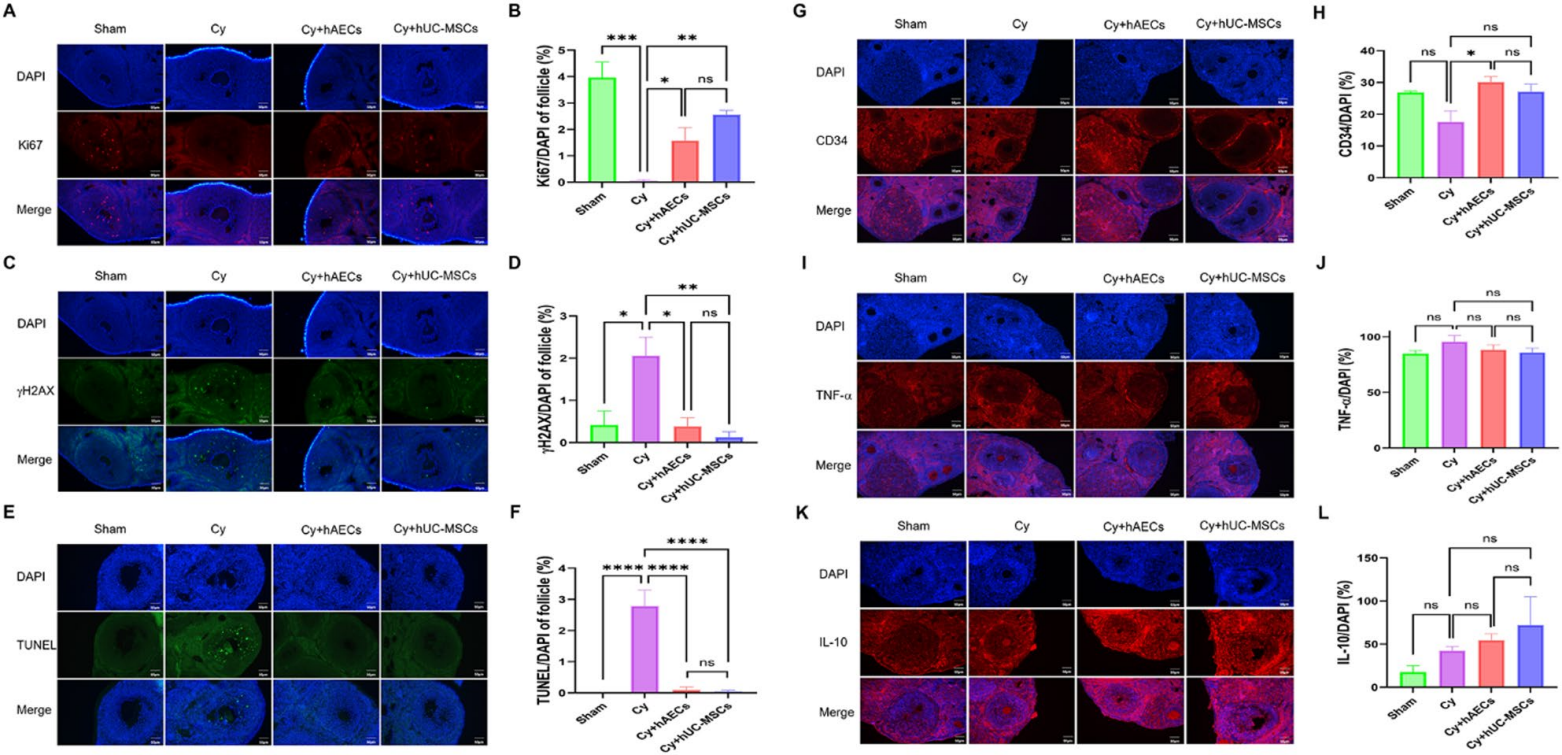
Effects of hAEC and hUC-MSC transplantation on the ovarian microenvironment after chemotherapy. **A**–**F** Representative images and quantitative analysis of Ki67 (proliferation), γH2AX (DNA damage), and TUNEL (apoptosis) in ovarian tissues across treatment groups. **G**–**L** Expression levels of CD34 (angiogenesis), TNF-α and IL-10 (inflammatory markers) in ovarian tissues from each group. Data are presented as means ± SEM (*n* = 5 mice per group, *P < 0.05; **P < 0.01; ***P < 0.001; ****P < 0.0001). Scale bars represent 50 μm

To comprehensively assess the therapeutic effects of hAECs and hUC-MSCs, we examined their impact on cellular proliferation, apoptosis, DNA damage repair, angiogenesis, and inflammatory responses in damaged ovaries. Results showed a significant decrease in the proliferation marker Ki67 in the Cy group, accompanied by elevated expression of the DNA damage marker γH2AX and an increased in TUNEL-positive apoptotic cells compared to Sham controls. Both hAECs and hUC-MSCs transplantation effectively restored cell proliferation (increased Ki67^+^ cells), reduced DNA damage (decreased γH2AX), and suppressed apoptosis (fewer TUNEL^+^ cells), with no significant difference observed between the two cell types (*P* < 0.05, Fig. 3A–F). Analysis of vascular and inflammatory maker expression showed a moderate decreased in CD34 expression in the Cy group, while the levels of the pro-inflammatory cytokine TNF-α and anti-inflammatory IL-10 were increased, though these changes did not reach statistical significance. Notably, hAEC transplantation significantly increased CD34 expression, whereas neither hAECs nor hUC-MSCs exerted a significant effect on the expression of inflammatory factors in ovaries (*P* < 0.05, Fig. 3G–L). These results demonstrate that both hAECs and hUC-MSCs similarly alleviate chemotherapy-induced ovarian damage, notably, hAECs exhibit a superior capacity to promote angiogenesis.

### Assessment of the retention and metabolic clearance rates of hAECs and hUC-MSCs in vivo

**Fig. 4:**
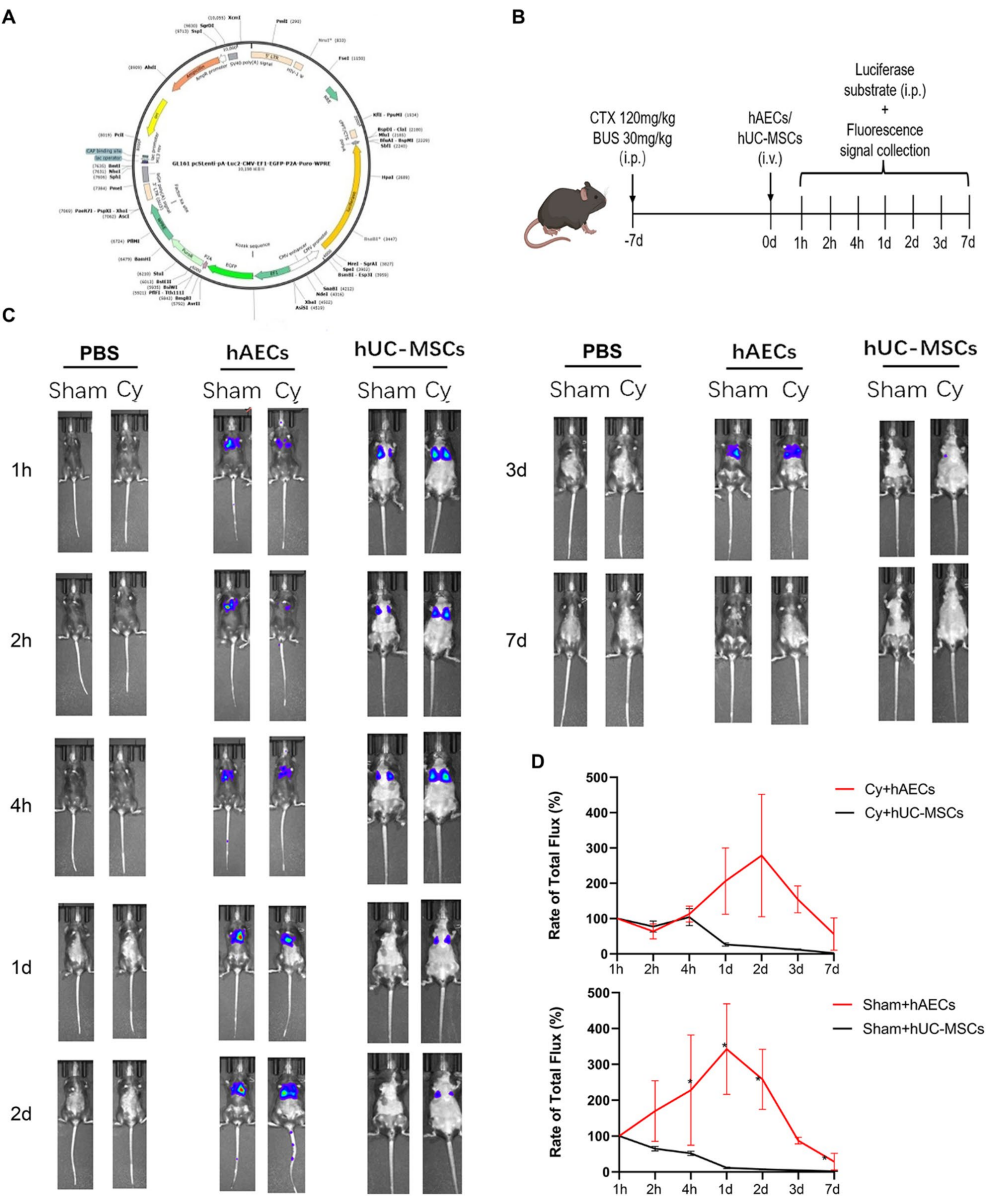
In vivo biodistribution and metabolic kinetics of hAECs and hUC-MSCs. **A** Schematic representation of the lentiviral vector constructed to express the luciferase reporter gene. **B** Schematic of the experimental setup for tracking the in vivo retention of lentivirus-labeled stem cells in mice. **C** Whole-body ioluminescence images of mice acquired at 1 h, 2 h, 4 h, 1 d, 2 d, 3 d and 7 d post-transplantation, visualized using the IVIS imaging system. **D** Quantitative analysis of fluorescence signals at the indicated time points after cell transplantation. Data are presented as means ± SEM (*n* = 4 mice per group, *P < 0.05; **P < 0.01; ***P < 0.001; ****P < 0.0001)

To compare the in vivo metabolic clearance rates of the two stem cell types, hAECs and hUC-MSCs were transduced with a lentivirus carrying a luciferase reporter gene. The labeled hAECs or hUC-MSCs were then transplanted via tail vein injection into both Sham and chemotherapy (Cy)-induced POI mice (Fig. 4A). At designated time point’s post-transplantation (1, 2, 4 h, and 1, 2, 3, 7 day), the luciferase substrate was administered intraperitoneally, and whole-body bioluminescence signals were captured using in vivo imaging (Fig. 4B). Longitudinal imaging analysis revealed that the transplanted hAECs or hUC-MSCs were predominantly located in the lungs, liver, and spleen in both Sham and Cy groups (Fig. 4C). The hAEC group showed an initial increase in bioluminescence intensity, followed by a gradual decrease. In contrast, the hUC-MSC group exhibited a consistent decline in signal over time (*P* < 0.05, Fig. 4D). These results indicate that hAECs have a significantly longer retention time in vivo compared to hUC-MSCs, suggesting distinct metabolic clearance rates between the two cell types.

### Comparison of antioxidant damage and anti-apoptosis effects between hAECs and hUC-MSCs

**Fig. 5:**
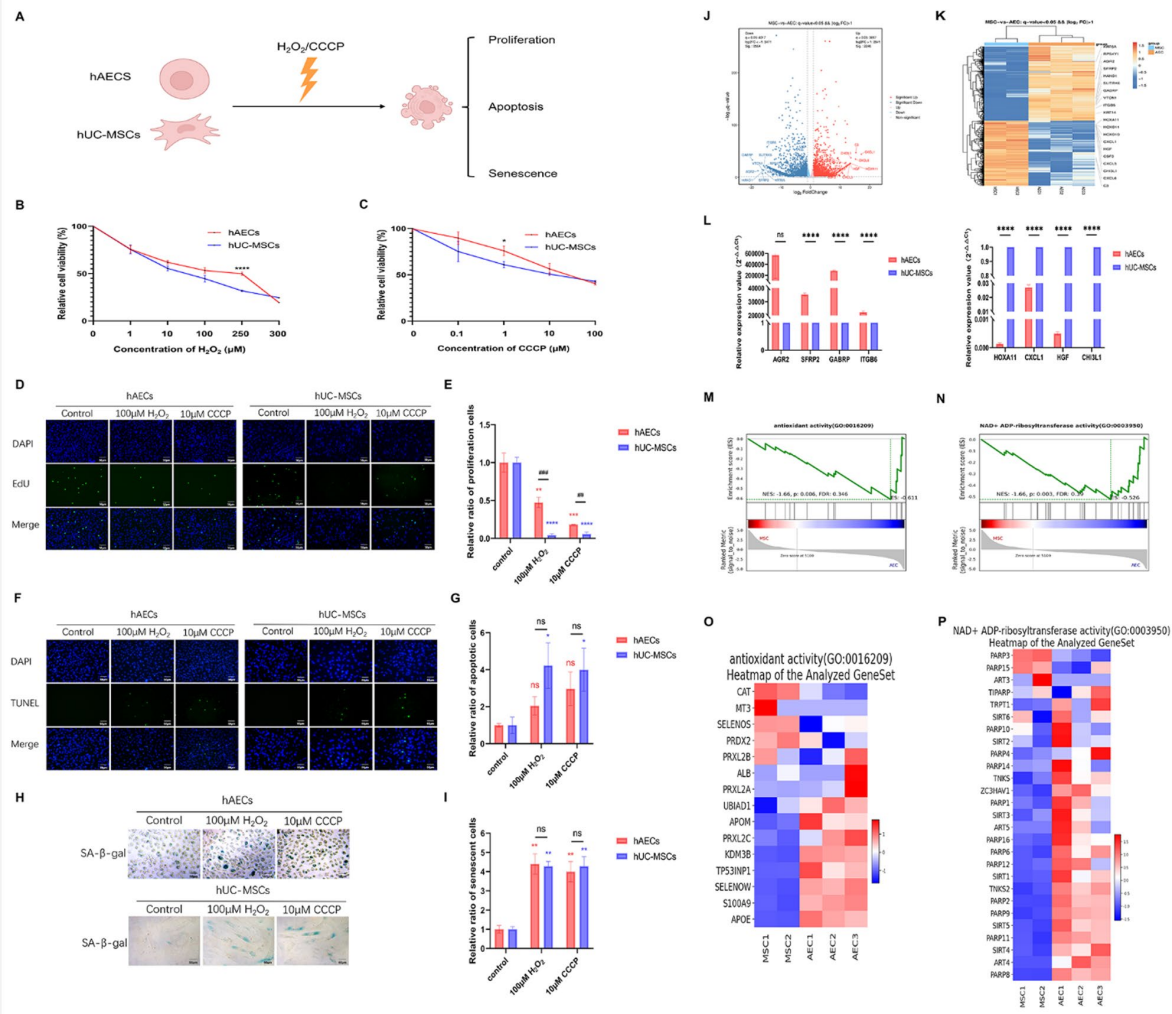
In vitro impact of adverse microenvironmental stressors on hAECs and hUC-MSCs. **A** Schematic representation of experimental design. **B**, **C** Cell viability of hAECs and hUC-MSCs following exposure to H_2_O_2_ and CCCP at various concentrations. **D**–**I** Quantification of proliferative, apoptosis and senescence rates in hAECs and hUC-MSCs treated with H_2_O_2_ and CCCP. **J**, **K** RNA-sequencing analysis of gene expression profiles in hUC-MSCs (*n* = 2) and hAECs (*n* = 3). **L** Validation of DEGs by qRT-PCR. **M**–**P** Gene set enrichment analysis and heatmap illustrating DEGs associated with antioxidant activity and NAD^+^ ADP-ribosyltransferase function. Data are presented as means ± SEM (*n* = 4–5 per group). *P < 0.05; **P < 0.01; ***P < 0.001;****P < 0.0001. Scale bars represent 50 μm

To compare the antioxidant damage and anti-apoptotic capacity of hAECs and hUC-MSCs in vitro, both cell types were exposed to oxidative stress (H_2_O_2_) and an apoptosis inducer (CCCP) to stimulate the hostile microenvironment following chemotherapy (Fig. 5A). CCK-8 assays showed a dose-dependent decrease in survival rates for both cell types, with hUC-MSCs exhibiting significantly higher sensitivity. Under high-stress conditions (250 µM H_2_O_2_ or 1 µM CCCP), hUC-MSCs showed markedly lower viability than hAECs (*P* < 0.05, Fig. 5B, C). EdU staining indicated that both H_2_O_2_ and CCCP suppressed proliferation in hAECs and hUC-MSCs, with a more pronounced inhibitory effect on hUC-MSCs (*P* < 0.05, Fig. 5D, E). TUNEL assays further revealed a significant increase in apoptosis rates in hUC-MSCs after oxidative or apoptotic stress, whereas hAECs remained largely unaffected (*P* < 0.05, Fig. 5F, G). Although both stressors promoted senescence in both cell types, no significant intergroup differences were observed (*P* < 0.05, Fig. 5H, I).

To explore the underlying molecular mechanisms, RNA-seq was performed on hAECs and hUC-MSCs. Comparative transcriptomic analysis identified 4810 DEGs, including 2246 upregulated and 2564 downregulated gene in hUC-MSCs relative to hAECs (Fig. 5J, K). Key DEGs, such as *AGR2*, *SFRP2*, *GABRP*, and *ITGB6* (downregulated), and *HOXA11*, *CXCL1*, *HGF*, and *CHI3L1* (upregulated), were validated by qRT-PCR (*P* < 0.05, Fig. 5L). Reactome enrichment analysis further indicated higher antioxidant activity and NAD^+^ ADP-ribosyltransferase activity in hAECs than in hUC-MSCs (Fig. 5M–P). These results suggest that hAECs exhibit stronger resistance to adverse microenvironments, which may be attributed to their distinct gene expression profiles.

### Distinct paracrine profiles underlie the differential repair mechanisms of hAECs and hUC-MSCs

**Fig. 6:**
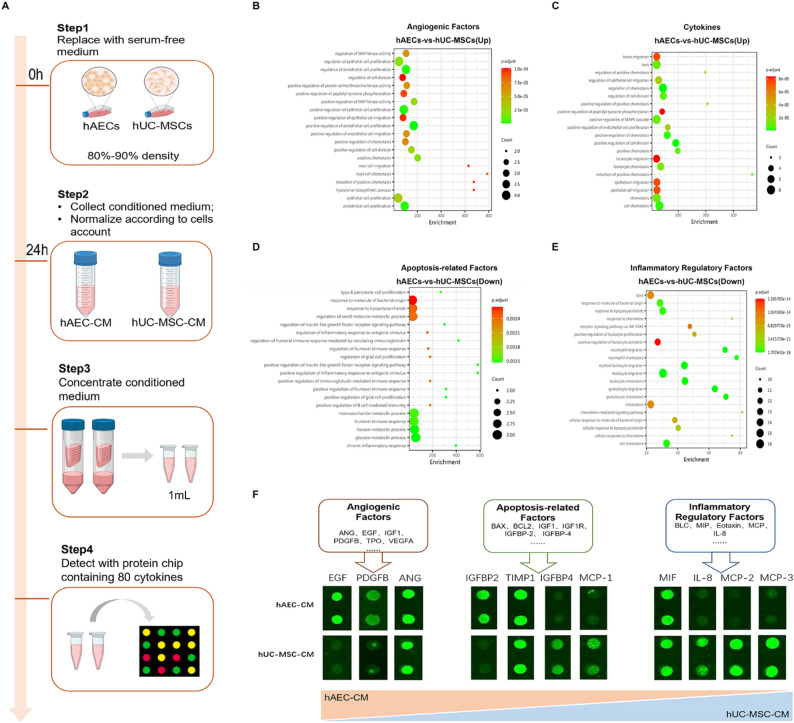
Analysis of paracrine factors in conditioned media from hAECs and hUC-MSCs. **A** Schematic representation of the experimental workflow for cytokine profiling in hAEC-CM and hUC-MSC-CM. **B**, **C** GO enrichment analysis of upregulated pathways related to angiogenesis and cytokine activity in hAECs compared to hUC-MSCs. **D**, **E** GO enrichment analysis of downregulated pathways associated with apoptosis and inflammatory regulation in hAECs relative to hUC-MSCs. **F** Protein microarray results displaying expression levels of selected cytokines in hAEC-CM and hUC-MSC-CM

To investigate the mechanistic differences in ovarian repair mediated by hAECs and hUC-MSCs, we analyzed their CM using protein microarrays (Fig. 6A). Secreted proteins were classified into four functional categories: angiogenic factors, apoptosis-related factors, inflammatory regulators, and other cytokines. hAEC-CM exhibited significantly higher levels of angiogenic factors, including regulators of endothelial/epithelial cell proliferation and chemotaxis mediators, as well as cytokines involved in MAPK cascade activation and cell division regulation (Fig. 6B, C). hUC-MSC-CM showed elevated expression of apoptosis-related factors, such as mediators of humoral immune response and IGF receptor signaling regulators, along with inflammatory modulators including leukocyte migration and chemotaxis factors (Fig. 6D, E).

**Fig. 7:**
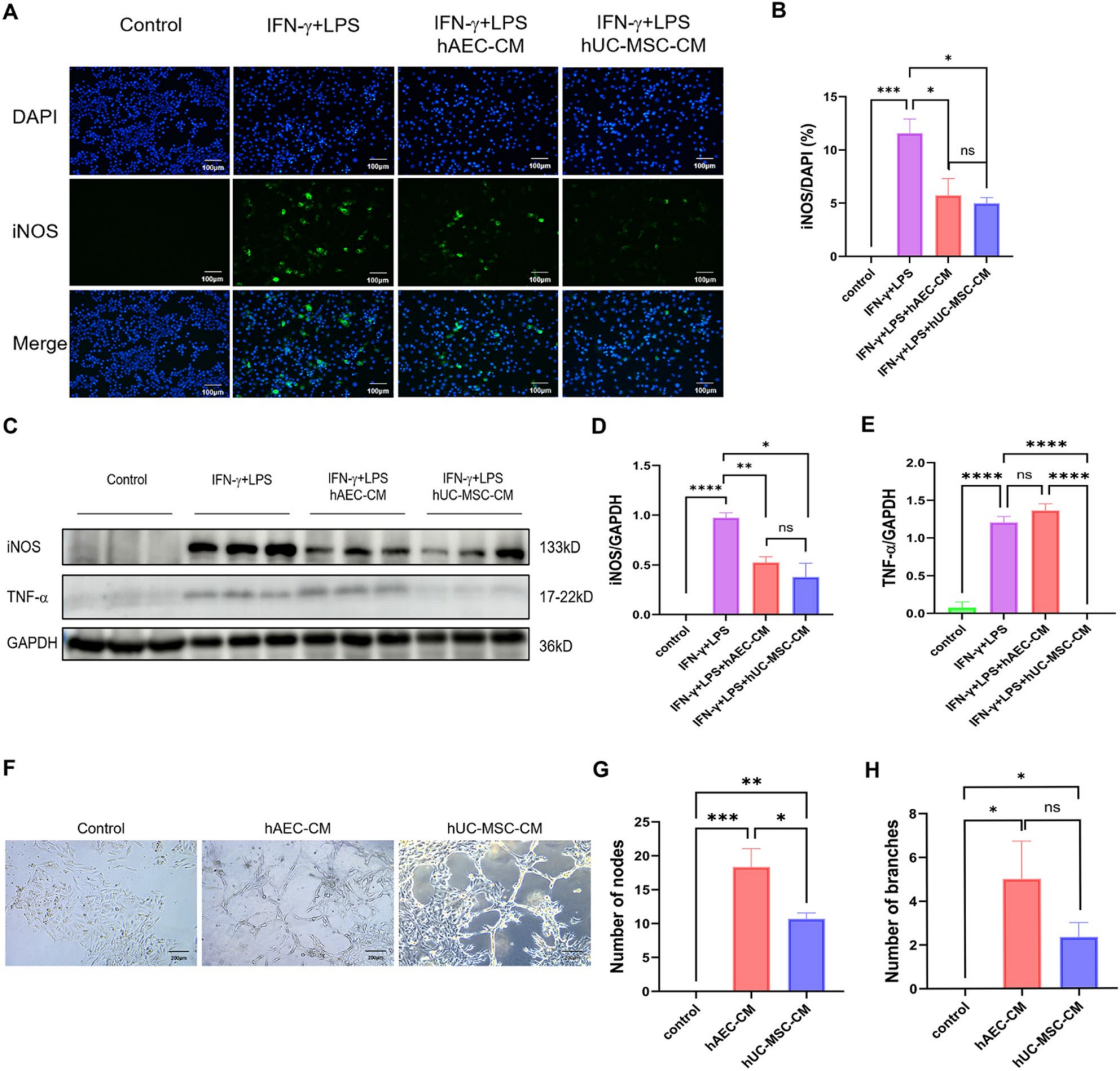
Effects of hAEC-CM and hUC-MSC-CM on inflammatory response and angiogenesis in vitro. **A** Representative immunofluorescence images showing iNOS expression in RAW264.7 macrophages stimulated with IFN-γ and LPS. **B** Quantitative analysis of iNOS expression in RAW264.7 cells across treatment groups (*n* = 4–5). **C**–**E** Protein expression levels of iNOS and TNF-α in different groups, as determined by western blotting (*n* = 3). **F** Tube formation assay of hUVECs treated with hAEC-CM or hUC-MSC-CM. **G**, **H** Quantification of tubular network nodes and branches in hUVECs following treatment with hAEC-CM or hUC-MSC-CM (*n* = 4–5). Data are presented as means ± SEM. *P < 0.05; **P < 0.01; ***P < 0.001;****P < 0.0001. Scale bars represent 100 and 200 μm

To further validate these findings, we compared the immunomodulatory and angiogenic capacities of hAEC-CM and hUC-MSC-CM in vitro. In an IFN-γ/LPS-stimulated RAW264.7 macrophage model (M1 polarization), both hAEC-CM and hUC-MSC-CM effectively suppressed iNOS expression. However, only hUC-MSC-CM significantly reduced TNF-α levels (*P* < 0.05, Fig. 7A–E). In hUVECs tube formation assays, both CMs enhanced tubular network complexity, increasing the number of nodes and branches. hAEC-CM produced significantly more nodes than hUC-MSC-CM and showed a trend toward increased branching, though this did not reach statistical significance (*P* < 0.05, Fig. 7F–H). These results demonstrate that hAEC-CM has enhanced angiogenic properties, whereas hUC-MSC-CM exhibits superior anti-inflammatory activity, reflecting their distinct paracrine mechanisms in ovarian function repair.

## Discussion

In this study, we systematically compared the regenerative mechanisms of two clinically relevant stem cell types, epithelium-derived hAECs and stroma-derived hUC-MSCs, in restoring ovarian function. In a mouse model of chemotherapy-induced POI, both cell types effectively improved ovarian function, as demonstrated by increased follicle counts and reduced fibrosis. Longitudinal in vivo imaging revealed similar biodistribution patterns between hAECs and hUC-MSCs; however, hAECs exhibited significantly prolonged retention, suggesting distinct metabolic properties. Under oxidative stress, hAECs demonstrated superior antioxidant capacity. Cytokine profiling showed that hAEC-CM was enriched in pro-angiogenic factors, while hUC-MSC-CM contained higher levels of immunoregulatory cytokines, a finding consistently supported by functional assays. These results provide important insights for cell-type-specific selection in cell-based therapies aimed at ovarian regeneration.

POI represents a chronic condition involving progressive ovarian injury, encompassing germ cell apoptosis, interstitial vascular degeneration, immune homeostasis disruption, and impaired follicular development [5]. Numerous studies have confirmed that stem cell transplantation can effectively restore ovarian function and enhance fertility in POI, primarily through the paracrine effects of the engrafted cells [24]. Nevertheless, comparative studies examining the regenerative capabilities and distinct advantages of hAECs versus hUC-MSCs are still scarce.

The biodistribution and metabolic kinetics of transplanted stem cells critically influence their therapeutic efficacy and safety. Non-invasive imaging techniques enable real-time tracking of hAECs and hUC-MSCs, providing insights into their migration and metabolic behavior. Current molecular imaging data indicate that stem cells exhibit organotropic distribution and time-dependent dynamics localization [25]. Consistent with prior reports [26, 27], intravenously infused stem cells primarily accumulated in the lungs, liver, and spleen. Notably, hAECs were initially detected in the lungs at 1 h post-injection and subsequently redistributed to the spleen and liver by day 1. In contrast, hUC-MSCs predominantly remained trapped in the lungs, with signal intensity gradually declining over time, indicating faster clearance. The differential first-pass retention and metabolic rates between the two cell types may be influenced by intrinsic cellular properties, such as cell size, which could represent a key determinant of their distinct in vivo.

In addition to their inherent properties, the capacity of stem cells to withstand damage also significantly influences their retention rates. To explore the differential stress resistance between these two types of stem cells, we conducted in vitro experiments. Combined with RNA-sequencing results, hAECs demonstrated superior antioxidative capacity compared to hUC-MSCs, which likely contributes to their enhanced ability to endure the harsh microenvironment in chemotherapy-induced POI mice.

Numerous studies have characterized the paracrine factors secreted by hUC-MSCs [28], identifying several important molecules implicated in regulating the functional remodeling of damaged ovaries [29]. Recent proteomic analyses have further revealed differences between these two cell types, both exhibiting promising angiogenic and anti-fibrotic potential [30]. In this study, we compared their cytokine profiles and found that hAEC-CM contained relatively higher levels of angiogenic factors, whereas hUC-MSC-CM was enriched in apoptosis-related factors and inflammatory regulators.

Stem cell therapy holds promise for treating POI, particularly due to its multi-mechanistic restorative effects, a feature unattainable with small-molecule drug interventions. Therefore, elucidating the repair characteristics of distinct stem cell types is essential for developing personalized treatments for ovarian dysfunction. In future clinical practice customized personalized stem cell therapy regimens based on the pathological profiles of POI patients may optimize therapeutic outcomes and minimize potential risks.

This study has several limitations that warrant consideration. First, although in vivo imaging whole-body bioluminescence signals was used to track stem cell migration and metabolic activity, ex vivo bioluminescence signals of ovaries was not performed. Further studies could focus on examining bioluminescence signals within isolated ovaries or employ advanced multimodal imaging techniques to precisely delineate cellular migration routes [31]. Second, conventional analyses inadequately resolve stem cell effects on stage-specific folliculogenesis from primordial to antral follicle. Integrated scRNA-seq with spatial transcriptomics would enable single-cell-level mapping of therapeutic responses across follicular stages [32]. Third, the commercial cytokine array used in this study was limited to detecting known cytokines and may missed lineage-specific secreted cytokines. Importantly, exosomes, as important components in the paracrine pathway of stem cells, play critical roles in functional repair of damaged tissues. Our current study has not conducted a detailed comparative analysis of the characteristics and cargo of exosomes from hAECs or hUC‑MSCs. Subsequently, the use of quantitative mass spectrometry to identify specific paracrine mediators secreted by these cells, as well as functional analysis of their exosome, is crucial for elucidating previously unrecognized repair mechanisms.

## Conclusions

Our results demonstrate that both hAECs and hUC-MSCs effectively restore ovarian function and preserve fertility in a chemotherapy-induced POI mouse model. However, these cell types exhibit distinct metabolic and paracrine characteristics. Specifically, hAECs show prolonged in vivo retention compared to hUC-MSCs, which may be attributed to their enhanced antioxidant capacity. In terms of paracrine function, hAECs possess superior pro-angiogenic activity, while hUC-MSCs display stronger immunomodulatory properties. These differential features offer valuable guidance for selecting appropriate stem cell-based therapies in POI patients.

## Supplementary Information

Below is the link to the electronic supplementary material.

Supplementary Material 1.

Supplementary Material 2.

## Data Availability

All the raw datasets generated during the current study are available from the corresponding author on reasonable request.

## Abbreviations

POI: Premature ovarian insufficiency
hAECs: Human amniotic epithelial cells
hUC-MSCs: Human umbilical cord mesenchymal stem cells
CM: Conditioned media
HRT: Hormone replacement therapy
PBS: Phosphate-buffered saline
FBS: Fetal bovine serum
PFA: Paraformaldehyde
BSA: Bovine serum albumin
GO: Gene Ontology
KEGG: Kyoto Encyclopedia of Genes and Genomes
HE: Hematoxylin and eosin
DAB: Diaminobenzidine
E2: Estradiol
AMH: Anti-Müllerian hormone
CCCP: Carbonyl cyanide 3-chlorophenyl hydrazone
Cy: Chemotherapy
TNF-α: Tumor necrosis factor-alpha
IL-10: Interleukin-10
