# Supplementary material for: Comparative evaluation of the therapeutic efficacy between human amniotic epithelial cells and human umbilical cord mesenchymal stem cells in premature ovarian insufficiency
Source: Stem Cell Res Ther. 2025 Dec 31;17:65. doi: 10.1186/s13287-025-04881-7 (PMC12866098; doi:10.1186/s13287-025-04881-7)
Supplement: Supplementary file 1 — Supplementary Material 1. [file 13287_2025_4881_MOESM1_ESM.pdf]

Supporting Information

Comparative Evaluation of the Therapeutic Efficacy between Human Amniotic Epithelial Cells and Human Umbilical Cord Mesenchymal Stem Cells in Premature Ovarian Insufficiency

Qinyu Zhang[1#], Jie Wang[1#], Zixin Cheng[1], Wenjiao Cao[1], Qiuwan Zhang[1,2]*, Dongmei Lai[1,2]*

1. The International Peace Maternity and Child Health Hospital, School of Medicine, Shanghai Jiao Tong University, Shanghai, China.

2. Shanghai Key Laboratory of Embryo Original Diseases, Shanghai, China.

[#]These authors contribute equally

*Corresponding Authors:

Dongmei Lai

E-mail: laidongmei@hotmail.com

Qiuwan Zhang

E-mail:zhangqiuwan@163.com

Membrane 1#

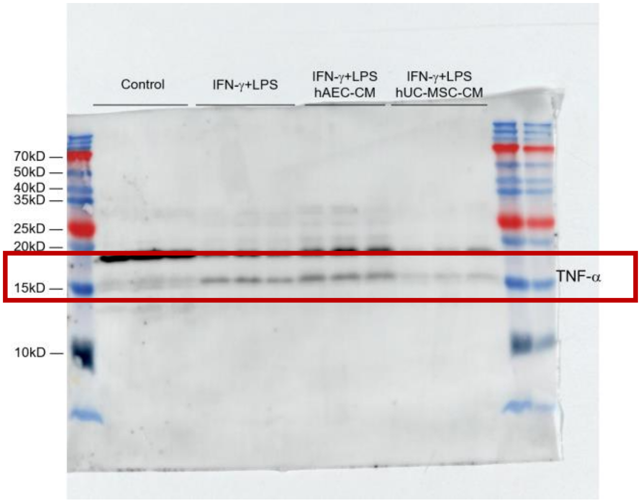

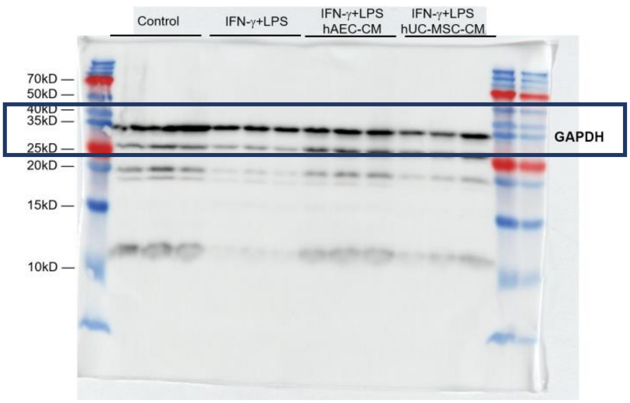

Membrane 2#

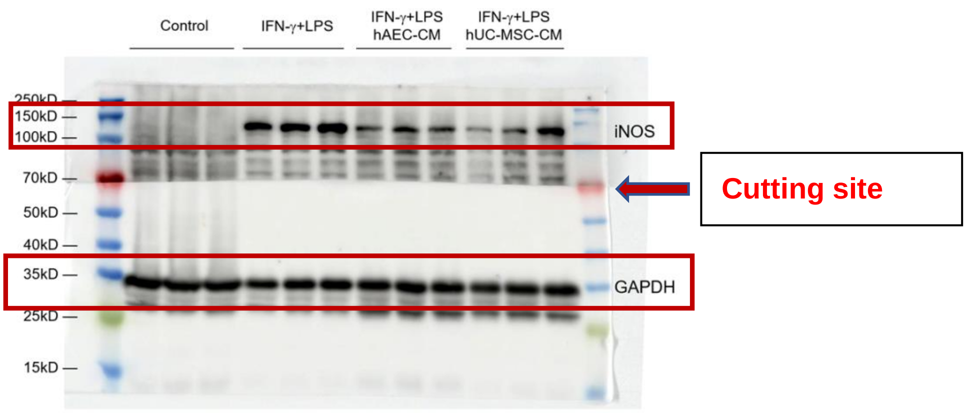

Figure S1. The full-length blot images showing the grouping of blots in Figure 7C.

Molecular weight marker for protein (kDa) indicated. Red frames indicate cropped parts used in figure 7C.
